# Class II Malocclusion Treatment by In-House Fabricated, Customized Fixed Functional Appliance in Growing Child

**DOI:** 10.1155/2022/8102482

**Published:** 2022-08-17

**Authors:** Swati Verma, Falguni Mehta, Mohammad Khursheed Alam, Harshikkumar Arvindbhai Parekh, Valai Kasim Shakeel Ahmed, Chhavi Jain

**Affiliations:** ^1^Community Healthcare Center, Department of Medical Health and Family Welfare, State Ministry of Health and Family Welfare, Government of Uttar Pradesh, Kanpur 208016, India; ^2^Department of Orthodontics and Dentofacial Orthopaedics, Government Dental College and Hospital, Ahmedabad 380016, India; ^3^Orthodontics, Preventive Dentistry Department, College of Dentistry, Jouf University, Sakaka 72345, Saudi Arabia; ^4^Department of Public Health, Faculty of Allied Health Sciences, Daffodil International University, Dhaka, Bangladesh; ^5^Department of Orthodontics, Ragas Dental College and Hospital, Chennai 600119, India; ^6^Hamdard Institute of Medical Sciences and Research, New Delhi 110062, India

## Abstract

Currently, wide arrays of fixed functional appliances are available for the correction of Class II malocclusion. The orthodontist must choose from these appliances depending on the mechanics, efficiency, and cost of the appliance. Fixed functional appliances may reduce the individual visits and hence the cost as compared to the removable appliances. Additionally, it may save the clinician's chair time. This report discussed the in-house laboratory fabrication and clinical procedure of customized fixed functional appliances by utilizing the readily available dental materials in the orthodontic clinical setting.

## 1. Introduction

Class II malocclusion is an anterior–posterior skeletal dysplasia with disto-occlusion. It is a broad and pervasive area of concern as 30% of all orthodontic patients seek treatment for this malocclusion [1]. The prevalence among global population is 18–34% [2, 3]. The northern states of Indian population have a 15% prevalence of Class II malocclusion [4]. Class II malocclusion can occur due to mandibular retrognathism, maxillary prognathism, or a combination of both. Mandibular retrognathia accounts for 75% of Class II malocclusion cases and is most dominant factor [5, 6]. The therapeutic intervention broadly depends on the growth status of the patient.

Considering the frequent encounter with Class II malocclusion in clinical set up, an orthodontist is always well equipped with the various treatment options like interception by functional therapy, camouflaging by extractions of premolars, and orthognathic surgery depending upon etiology of Class II malocclusion, the skeletal age, gender, the compliance of the patient and cost–benefit ratio [7–11].

Functional appliances are growth modification appliances used during the growing stage for skeletal correction. There are two broad categories of functional appliances: removable and fixed. Rigid, hybrid, and flexible types of fixed functional appliances are all commercially available subcategories. Available literature reported that Rigid fixed functional appliances (FFA) showed the highest promising results in Class II malocclusion among growing patients [12, 13]. This report discussed the in-house laboratory fabrication and clinical procedure of customized fixed functional appliance, Falguni Mehta's Mesial Jet (FM2 JET), by utilizing the readily available dental materials in the orthodontic clinical setting.

## 2. Diagnosis and Etiology

**Figure 1:**
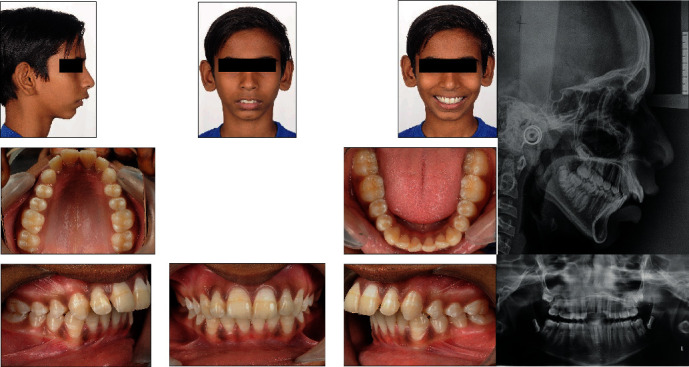
Pretreatment photographs and radiographs.

A 13-year-old male patient presented to the department with a chief complaint of prominent upper front teeth and difficulty in closing lips. Patient's general health was good with no past medical history. Extraoral examination showed convex profile with severely retruded chin button, incisal display at rest, and potentially competent lips. Smile aesthetics revealed average gingival display (Figure 1). No abnormal finding was detected on temporomandibular joint examination. The patient had 11 mm of overjet and average overbite with flat curve of spee. The gingival and periodontal statuses were good with no signs of inflammation. Angle Class II molar and canine relationship was present bilaterally. The upper and lower dental midline were coincident relative to facial midline. Distobuccal rotation with 14, 15, 24, and 25 and mesiolingual rotation with 32 and 42 were found. Model analysis revealed a space availability of 0.5 mm in maxillary arch. The pretreatment panoramic radiograph revealed a full complement of permanent teeth and no aberrations in the surrounding structures or regions (Figure 1). Third molar tooth buds were evident in all four quadrants. The cephalometric analysis showed a Class II division 1 skeletal pattern due to retrognathic mandible with vertical growth tendency and cervical vertebral maturation index (CVMI) stage III as summarized in Table 1. The lower incisors were proclined. Soft tissue analysis revealed upturn nose with average nasolabial angle and protrusive lower lip.

## 3. Treatment Objectives

The treatment objectives were to (1) achieve correction of Skeletal Class II relationship by functional jaw orthopedics, (2) achieve alignment and leveling of upper and lower arches, (3) achieve Class I molar and canine relationship, (4) achieve ideal overjet and overbite, (5) achieve average soft tissue facial profile, and (6) gain ideal occlusion with good intercuspation.

## 4. Treatment Plan

The patient is adolescent with deficient mandible. So myo-functional therapy could be applied with good expected results. Additionally, the patient's parents opted for nonsurgical, less time-demanding, and less bulky functional appliance with minimal visibility. So, we decided to go for one phase myofunctional therapy with in-house-fixed functional appliance along with the multibracket system. We made our FFA a rigid appliance so that it can achieve desired combined skeletal and dentoalveolar correction.

## 5. Appliance Principle and Fabrication

**Figure 2:**
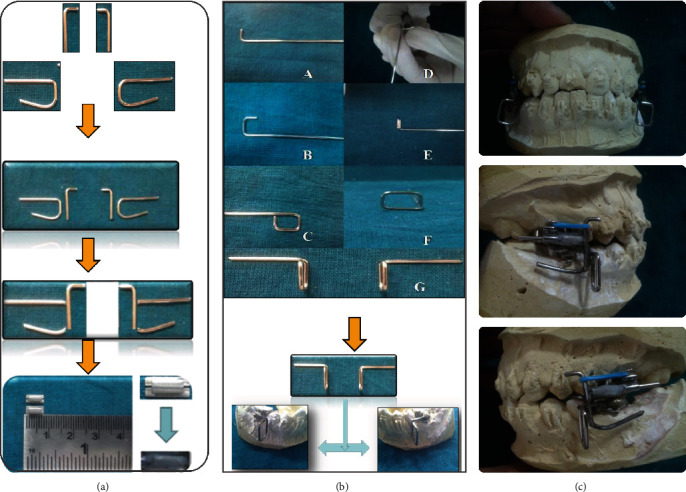
Schematic representation of steps in fabrication of appliances: (a) upper assembly, (b) lower assembly, and (c) in-house MARA.

Rigid fixed functional appliance has a restricting effect on the maxilla similar to the headgear effect and a protrusive effect on the mandible [14]. When the upper and lower working models were kept in edge-to-edge incisal relationship, the upper assembly had an L-shaped hook with a 3 mm anterior projection and a U-shaped assembly with a 15 mm upper leg that is bent at 90° and continues vertically to the cervical to lower border of the lower buccal tube. Distally, marked and an acute rounded bend was made. Lower leg length was maintained at 3/4ths of the length of the upper leg. L-shaped hooks were then spot-welded on both sides of the buccal side of the vertical leg of the U-shaped assembly to provide stability. Finally, both components were soldered together and polished using a polishing rubber disc to create an upper assembly that resembles a “Swan” (Figure 2(a)).

**Figure 3:**
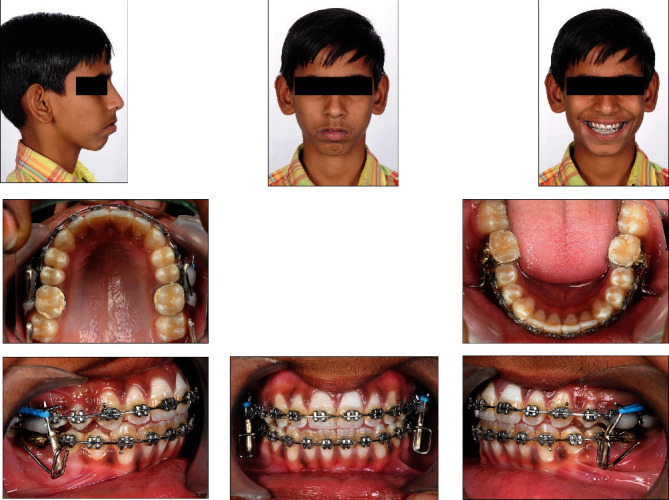
With in-house fabricated*, customized FM2 JET appliance* photographs and radiographs.

Lower assembly was fabricated sequentially as a rectangle with a length of 4 mm and a width of 5 mm formed from 0.036 inches stainless-steel wire in such a way that it lies buccally perpendicular to the buccal surface of the teeth and the retentive arm exits at the occluso-mesial angle of the rectangle. The retentive arm was then bent distally 90° and adapted to fit well over buccal surface of the lower molar bands. The dental plaster was used to block a base arch wire 0.022 slot. Following that, the lower assembly was stabilized and soldered on both sides (Figure 2(b)). The assembly was then removed from the working dental cast and polished. Oval buccal tubes with internal diameter of 0.027 × 0.050-in were used to fabricate customized inserts for activation of the upper assembly. The undercuts in oval buccal tubes were filled with composite material and polished to provide a self-cleansing region (Figures 2(a) and (c)). The insert's size usually determined by the amount of horizontal advancement necessary in the patient. The upper bands were then cemented bilaterally, followed by the lower assembly. Time was allowed for the cement to set. The customized inserts were inserted into the upper assembly, and then the upper assembly was secured to the maxillary first molar's headgear tube. The appliance was positioned bilaterally to overcorrect the Class II occlusion to an edge-to-edge incisor relationship (Figure 3). If the patient attempted to close his or her mouth in the retrusive position, the upper assembly's inclines guided the jaw forward, resulting in growth modification.

## 6. Treatment Progress

The treatment was initiated with banding of molars and placement of preadjusted edgewise 0.022-in slot brackets with McLaughlin, Bennett, Trevisi (MBT) system prescription to the maxillary and mandibular teeth. A 0.016-in nickel–titanium wire was engaged as the initial aligning archwire for leveling and alignment. Then archwires were sequentially progressed to 0.017 × 0.025-in nickel–titanium archwire, 0.019 × 0.025-in nickel–titanium archwire, and 0.019 × 0.025-in stainless-steel archwire in both the arches. The patient was reluctant to any kind of surgical intervention or extraction. Therefore, authors had to rely on progressive inter-proximal reduction (IPR) and segmental torque application with caution for lower incisor inclination control. A 20° torque was incorporated in 0.019 × 0.025-in stainless-steel archwire in relation to lower arch with 2 mm of progressive interproximal stripping. No curve of spee was incorporated as patient had a vertical growth tendency. A figure of eight with a ligature wire was used to consolidate both upper and lower arches and distal ends of arch wires were cinched back. After completion of alignment and leveling in 4 months, the sagittal functional correction was initiated. Working models with molar bands of upper and lower arches were made. The patient was provided postinsertion instructions.

After 10 months in situ, the appliance was removed. Then, the final finishing and detailing of the occlusion were adjusted for two months before debonding. The final detailing of the occlusion was achieved with 0.017 × 0.025-in nickel–titanium in conjunction with night wear of light elastics with short Class II vectors. The retension phase of 11 months included wear of a maxillary removable Hawley retainer with anterior inclined bite plane and fixed lingual retainer in relation to mandibular arch. The patient was instructed to wear Hawley's Appliance full time for six months followed by night wear. The total duration of the treatment was 28 months. The patient's compliance was good throughout the treatment.

## 7. Treatment Results

**Figure 4:**
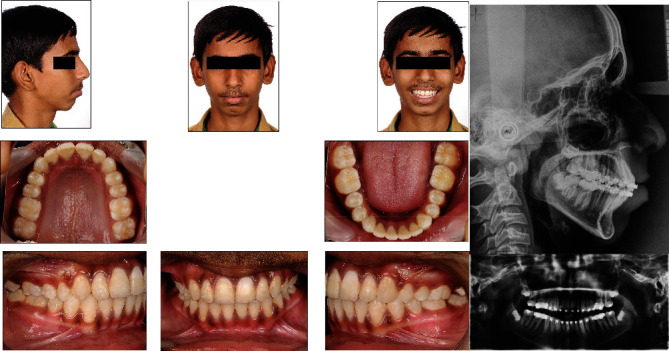
Posttreatment photographs and radiographs.

**Figure 5:**
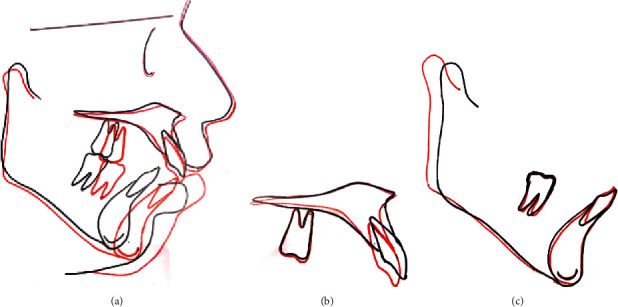
(a) Overall structural superimposition; regional (b) maxillary; and (c) mandibular structural superimposition.

The anteroposterior dysplasia was managed successfully and a large overjet was corrected with single-phase myofunctional therapy with In-house fabricated FM2 JET. The positive forward growth and augmentation of mandibular length were observed at condyle with increase in angle between a sella-nasion plane and point B (SNB) and Go-Pg. There was growth restriction of maxilla evident in angle between a sella-nasion plane and point A (SNA). The mandibular plane angle was maintained throughout the course of therapy. The dentoalveolar objectives achieved were, a well interdigitated buccal occlusion with Class I canine and molar relationship bilaterally, a well aligned and coordinated upper-lower dental arches with coinciding midlines, and a functional occlusion with stable posterior support and correct anterior guidance, with ideal overjet and overbite. Mandibular incisors showed that dentoalveolar inclination was maintained and the roots were well centralized within the cortices in posttreatment with average interincisal angle and average inclinations of the maxillary incisors in favorable position. All the treatment objectives were accomplished. Posttreatment photographs depict an aesthetically pleasing, well-balanced harmonious facial proportion. (Figure 4) except the chin button which was optimally protracted from pretreatment (Figure 1). Table I demonstrates the pre- and post-treatment cephalometric values. The posttreatment panoramic radiograph and cephalogram illustrate a root parallelism with no root resorption or marginal bone loss, and a harmonious maxillomandibular relationship. Overall and regional superimposition of the pretreatment and posttreatment cephalometric tracings showed treatment-related and growth-related correction in the sagittal skeletal relationship as well as in the soft tissue facial profile (Figure 5). Although mild soft tissue irritation under the appliance was monitored for the first few days after installation of appliance, the patient and his parents were pleased with the treatment results.

## 8. Discussion

This designed appliance was an active type of fixed functional appliance that utilizes a mechanically rigid mechanism to retain the mandible forward. Due to the continuous mandibular anterior repositioning, remodeling of the condyle occurs, resulting in forward placement of mandible and improving the facial proportions. Rigid fixed functional appliance has a restricting effect on the maxilla similar to the headgear effect and a protrusive effect on the mandible [14]. The vector of force exerted on the maxillary incisors and molars was distal. As the lower assembly was limited to molar area, the better control was reported in relation to lower incisors than with other fixed functional appliances.

Class II malocclusion is a sagittal discrepancy that may occur due to deficient mandible, prognathic maxilla, or a combination of both. According to McNamara, the most common etiologic feature of Class II malocclusion is a retrognathic mandible [15]. The prevalence of Class II malocclusion and its traits is 4.5–26% in Asian population [16]. In India, Class II malocclusion has been reported to be relatively more prevalent in north Indian population [4]. A great deal of contention is always surrounded by the determination of the best successful approach to adopt in the treatment of growing patients with Class II malocclusions [12]. Extraoral headgears can be employed in early mixed dentition stage if malocclusion is due to prognathic maxillae. It requires patient compliance. In patients with normal maxillary growth development and inhibited mandibular growth, the aim of the treatment should be to stimulate mandibular growth and to modify growth pattern with functional appliances.

Our patient was at pre-pubertal stage, ideal for functional appliance to bring about higher skeletal changes for Class II correction. The patient exhibited Class II malocclusion with retrognathic mandible and had vertical growth tendency so high-bite functional appliance might have been proved ideal choice. But on demonstrating the design and precautions of the removable appliance, the parents declined that option and demanded for less bulky appliance with no palatal attachment or surgical intervention. With these prerequisites, the available options got limited. With removable functional appliances, compliance exhibits as a crucial factor. Sahm et al. reported that most patients fulfill no more than 50% of the requirements concerning daily wear, and that was the major concern of the parents in our case [17]. Fixed functional appliances are much more efficient in stimulating skeletal mandibular growth than removable orthodontic appliance [18, 19]. In addition, the removable appliances are bulkier. Batista et al. also demonstrated that there were no significant treatment outcome differences between single-phase and two-phase myofunctional therapy [20]. Cost-related drawbacks of the two-phase approach, according to O Brien, include additional burden for the patient in terms of attendance, expenditures, and chairside treatment time, and a sustained duration of total treatment [21].

While the mandibular anterior repositioning appliance (MARA) does not utilize telescopic tubes or springs to permanently link the jaws, it does allow for greater mandibular movement. That is why we selected this fixed appliance for our patient and fabricated it in-house using readily used orthodontic materials [11]. The lower horizontal arm of maxillary assembly of the in-house FM2 JET appliance also prevents the closure of mandible in Class II relationship. The habitual training of constant anterior repositioning of the mandible developed a conducive environment for the mandible to grow in the desired direction during growing stage. In our patient, the objective was to promote positive remodeling in relation to the patient's facial skeleton. This correction was achieved by length augmentation of mandible and achieving harmonious balance between facial skeleton and neuromuscular adaptation. By harnessing the forces of the orofacial musculature, the appliance advanced the mandibular arch in a favorable direction. An adequate length of the vertical arm of the maxillary assembly assures that, even in the relaxed freeway space position, the mandible remains in the new desired position as a result of the neuromuscular reprogramming of the masticatory system [18]. Vertical control is critical in patients with a hyperdivergent pattern. Due to the patient's refusal of any palatal attachment or surgical treatment, we were confined to install a fixed functional appliance without transpalatal and lingual arch or reinforced anchorage by miniscrews. The treatment outcome was largely due to the successful redirection of patient's muscular forces by this appliance along with consolidated arches.

As illustrated in Figure 4, this appliance produced a head gear effect” in the maxillary arch and significant horizontal advancement of the mandible with well-supported posterior occlusion in the anteroposterior plane. The ramal height had also been increased in the vertical direction. It had improved the anterior and posterior facial height ratios proportionally, contributing to more harmony in the skeletal relationship.

Furthermore, the appliance was limited to the molar region, which resulted in a series of beneficial outcomes in our case. The appliance's placement in the molar area transmitted muscular forces to the area closest to the center of resistance in the maxillae and mandible. Due to the increased distance between the point of application of transmitted forces and the lower anterior region, compared to telescopic fixed functional appliances, the detrimental effects on the labial cortex and dentoalveolar segment were negligible. In addition, the cinched back and consolidated lower arch with 20° torque and progressive IPR collectively prevented inadvertent proclination of lower incisors. The therapeutic masticatory forces were transmitted to the molars and premolars with good cortical bony support and dissipated in equilibrium to entire dentition. Additionally, an appliance with this design enables the use of a fixed functional appliance in tandem with an orthodontic fixed appliance, therefore expediting the treatment outcome.

The advantages of this indigenous appliance can be streamlined according to the biomechanical advantage, clinician–patient-related and the cost–benefit ratio. The biomechanical basis was developed to ensure that the anticipated extraoral and intraoral effects materialized as planned. The appliance was not connected intermaxillary in design. As a result, it was convenient for the patient to open and close his mouth. This may aid in proper maintenance of oral hygiene, speech, and eating habits among patients. Psychologically, it helped as well, as the appliance was not grossly visible and was confined to relatively posterior region.

### 8.1. Limitations

This reported case could have these limitations. To begin, the patient may take the first few days to adapt just like any other functional appliance. Secondly, one school of thought may cast doubt on the necessary laboratory process. However, the authors believe it is advantageous since the proposed fixed functional appliance can simulate biomechanically sound clinical outcomes. It is helpful in individual dental setup especially in poor resource countries where the laboratory support is inconsistent. Further, well-designed prospective comparative clinical trial and research projects with good sample size are suggested for future implications. The chin position was still retrognathic due to hyperdivergent growth pattern. Therefore, therapeutic advancement genioplasty would be considered once the growth cease.

This case report demonstrates the use of this appliance to utilize the pubertal stage to manage a patient with Skeletal Class II malocclusion with severe overjet and hyperdivergent growth pattern to limit the morbidity of malocclusion to obtain a good skeletal and dentoalveolar results with favorable neuromuscular adaptation.

## 9. Conclusion

This case demonstrates an efficient and successful approach of growth modification for the correction of Class II malocclusion using an in*-house* fabricated, customized FM2 JET appliance.

## Figures and Tables

**Table 1:** Comparing pretreatment and posttreatment cephalometric values.

Variable	Pretreatment	Posttreatment	Norms
SNA (deg.)	83	81	82.0 ± 2
SNB (deg.)	72	76	80.0 ± 2
ANB (deg.)	10	5	2 ± 2
Beta angle (deg.)	22	33	Class I: 27–35
			Class II: <27
			Class III: >35
Wits appraisal (mm)	10	0.6	−2.4 ± 2.2
Mandibular ramus length (Ar-go)	48	52	51.34 ± 5.15
Mandibular body length (go-Pg)	76	85	82.20 ± 6.31
Upper incisor to SN (deg.)	114	102	102 ± 5.5
Upper incisor to NA (deg./mm)	25/4	21.37/5	4.3 ± 2.7
Lower incisor to NB	34/6	35/7	4.0 ± 1.8
Interincisal angle	110	112	130 ± 6.0
Lower incisor to mandibular plane	100	100	101.30 ± 9.19
Overjet (mm)	11	2	2 ± 2.0
Overbite (mm)	1.3	1.1	2 ± 2.0
FMA (deg.)	33	33	24 ± 4.5
Upper lip to E plane (mm)	4.96	1.31	−6 ± 2.0
Lower lip to E plane (mm)	5	6	−2 ± 2.0
Nasolabial angle	94	100	102 ± 8.0

## Abbreviations

FFA: Fixed functional appliances
FM2: Jet, Falguni Mehta's mesial jet.
